# Reducing unwanted trauma memories by imaginal exposure or autobiographical memory elaboration: An analogue study of memory processes

**DOI:** 10.1016/j.jbtep.2010.12.009

**Published:** 2012-12

**Authors:** Anke Ehlers, Jana Mauchnik, Rachel Handley

**Affiliations:** King’s College London, Department of Psychology (PO77), Institute of Psychiatry, De Crespigny Park, London SE5 8AF, UK

**Keywords:** Trauma memories, Priming, post-traumatic stress disorder, Reexperiencing, Evaluative conditioning

## Abstract

Unwanted memories of traumatic events are a core symptom of post-traumatic stress disorder. A range of interventions including imaginal exposure and elaboration of the trauma memory in its autobiographical context are effective in reducing such unwanted memories. This study explored whether priming for stimuli that occur in the context of trauma and evaluative conditioning may play a role in the therapeutic effects of these procedures. Healthy volunteers (*N* = 122) watched analogue traumatic and neutral picture stories. They were then randomly allocated to 20 min of either imaginal exposure, autobiographical memory elaboration, or a control condition designed to prevent further processing of the picture stories. A blurred picture identification task showed that neutral objects that preceded traumatic pictures in the stories were subsequently more readily identified than those that had preceded neutral stories, indicating enhanced priming. There was also an evaluative conditioning effect in that participants disliked neutral objects that had preceded traumatic pictures more. Autobiographical memory elaboration reduced the enhanced priming effect. Both interventions reduced the evaluative conditioning effect. Imaginal exposure and autobiographical memory elaboration both reduced the frequency of subsequent unwanted memories of the picture stories.

## Highlights

► We investigated cognitive mechanisms of interventions that reduce reexperiencing after trauma. ► Neutral objects shown in traumatic picture stories were later preferentially identified and evaluated negatively. ► Memory elaboration balanced perceptual priming for objects from traumatic and neutral stories. ► Imaginal exposure and autobiographical memory elaboration led to more balanced emotional evaluations. ► Interventions may prevent selective perceptual or emotional responses to trauma reminders.

## 1 Introduction

Unwanted distressing trauma memories are a core symptom of post-traumatic stress disorder (PTSD, American Psychiatric Association, 1994). A range of therapeutic interventions are effective in reducing such unwanted memories (for reviews see Bradley, Greene, Russ, Dutra, & Westen, 2005; Bisson et al., 2007). These include imaginal reliving of the trauma (Foa & Rothbaum, 1998) and cognitive interventions designed to increase the elaboration of the traumatic experience in terms of linking it with its context of other autobiographical information (Ehlers & Clark, 2000). This raises the question of what memory processes are involved in unwanted memories of trauma and their successful treatment.

This study focuses on two memory processes that may be involved in the triggering of unwanted trauma memories: perceptual priming and evaluative conditioning. Interest in perceptual priming was generated by clinical observations suggesting that a particularly wide range of stimuli can trigger unwanted trauma memories (e.g., Brewin, Dalgleish, & Joseph, 1996; Foa, Steketee, & Rothbaum, 1989). Interview and questionnaire studies suggested that triggers are often perceptually similar to the intrusive content or to stimuli that signalled the onset of these moments from the trauma (Ehlers, Hackmann, & Michael, 2004; Ehlers et al., 2002; Michael, Ehlers, Halligan, & Clark, 2005; Southwick et al., 1993). For example, a patient with PTSD kept seeing headlights coming towards him, just like he had seen them shortly before his head-on car crash. Observations in therapy suggested that these intrusions were often triggered by round patches of light on a dark surface, (e.g., a patch of sunlight on a lawn, white spots on a dark cloth). Ehlers and Clark (2000) suggested that the easy triggering of reexperiencing in PTSD by perceptually similar cues is in part a function of *strong perceptual priming* for stimuli that occurred shortly before and during the traumatic event.

Some studies have tested the hypothesis that stimuli that are associated with the trauma are more strongly primed in people with PTSD than in those without PTSD. Participants encoded trauma-related and control stimuli (mainly words or sentences) and priming was tested later with word-stem completion or perceptual identification tasks. The results mostly support the hypothesis of greater perceptual priming for material associated with the trauma in people with PTSD compared those without PTSD (Amir, Leiner, & Bomyea, 2010; Amir, McNally, & Wiegartz, 1996; Ehring & Ehlers, in press; McNally & Amir, 1996; Michael, Ehlers, & Halligan, 2005, but see McNally & Amir, 1996 for negative results). Priming for trauma-related words predicted PTSD severity 6 months later (Ehring & Ehlers, in press; Michael, Ehlers, & Halligan, 2005; Michael, Ehlers, Halligan, & Clark, 2005). These experiments investigated post-trauma priming and thus supported a role of priming in the maintenance of intrusive memories and generalization of triggers, but they did not investigate perceptual priming during the trauma.

Experimental analogue studies provided initial support for the role of perceptual priming in the development of analogue intrusive trauma memories in healthy controls (Arntz, de Groot, & Kindt, 2005; Ehlers, Michael, Chen, Payne, & Shan, 2006; Michael & Ehlers, 2007). For example, Ehlers et al. (2006) developed an experimental paradigm to study visual perceptual priming for stimuli that occur in a traumatic context. The paradigm investigates priming for neutral objects that occur just before something “traumatic” happens. Participants watch a series of “traumatic” and neutral picture stories. The content of the first picture is unemotional. It contains neutral preceding stimuli (e.g. a cushion) for which memory is later tested. During the second picture, either something “traumatic” (e.g., a man being attacked with a knife) or something neutral happens. The last picture focuses on the outcome of the story for the main character (e.g., the attacked man is being decapitated). In accordance with the enhanced perceptual priming hypothesis, the results showed that neutral stimuli preceding a “traumatic” event showed enhanced perceptual priming and predicted intrusive memories (Ehlers et al., 2006).

Michael and Ehlers (2007) replicated these results with a somewhat modified set of picture stories and further showed that a post-“trauma” experimental manipulation designed to promote autobiographical elaboration of the memory for the picture stories reduced the enhanced perceptual priming effect and the relative probability of subsequent reexperiencing symptoms. This intervention was modeled on procedures used in Cognitive Therapy for PTSD (Ehlers & Clark, 2000; Ehlers et al., 2003; Ehlers, Clark, Hackmann, McManus, & Fennell, 2005) that involve accessing the most traumatic moments from the trauma and their meanings, and then explicitly linking them with other information from the patient’s experience (or from cognitive restructuring) that give these moments a less threatening meaning.

Other memory processes of interest in the triggering of unwanted trauma memories are learned associations (e.g., Ehlers & Clark, 2000; Foa et al., 1989; Keane, Zimering, & Caddell, 1985). It has been suggested that during trauma fear responses become associated with stimuli that are present at the time, and subsequently generalize more broadly to stimuli and situations that resemble the original trauma (Pavlovian conditioning, e.g. Foa et al., 1989; Keane et al., 1985). Several studies demonstrated that trauma survivors with PTSD show heightened physiological reactivity to trauma reminders compared to traumatized and non-traumatized controls (see Pole, 2007, for a review). In addition, learned S–S associations may play a role (Ehlers & Clark, 2000). More generally, there is a large literature on evaluative conditioning (for reviews see De Houwer, Thomas, & Baeyens, 2001; Hofmann, De Houwer, Perugini, Baeyens, & Crombez, 2010). Evaluative conditioning refers to a change in the valence of a stimulus that is due to the pairing of that stimulus with another positive or negative stimulus. Evaluative conditioning has been shown with explicit measures of valence (e.g. ratings of how much the individual likes the stimulus) as well as implicit measures (e.g. startle responses) (Hofmann et al., 2010). Neutral stimuli that people perceive in the context of trauma may thus acquire negative valence via evaluative conditioning and may therefore become triggers of negative affect and unwanted trauma memories in its aftermath. Interestingly, the literature also shows that evaluative conditioning is modifiable by subsequent intervention such as extinction training (Hofmann et al., 2010).

The present experimental analogue study used Michael and Ehlers’s (2007) paradigm to test whether priming for stimuli that occur in the context of trauma and evaluative conditioning may play a role in the therapeutic effects of imaginal exposure and autobiographical memory elaboration on subsequent unwanted trauma memories. The present experiment examined the following hypotheses:

*Hypothesis 1 (Enhanced perceptual priming*): On the basis of studies by Ehlers et al. (2006) and Michael and Ehlers (2007), it was expected that neutral stimuli that immediately precede a “traumatic” picture will be more strongly primed than neutral stimuli that precede a neutral picture.

*Hypothesis 2 (Effects of elaboration on enhanced priming):* On the basis Michael and Ehlers’s (2007) findings, it was expected that post-trauma autobiographical memory elaboration, but not imaginal exposure or control tasks designed to prevent processing of the traumatic material, will reduce the enhanced priming effect.

*Hypothesis 3 (Evaluative conditioning).* On the basis of studies on evaluative conditioning (De Houwer et al., 2001; Hofmann et al., 2010), it was expected that participants will dislike neutral stimuli that immediately precede “traumatic” pictures to a greater extent than neutral stimuli that precede neutral pictures.

*Hypothesis 4 (Intervention effects on evaluative conditioning).* On the basis of research showing that evaluative conditioning can be modified by subsequent intervention (Hofmann et al., 2010), it was expected that the evaluative conditioning effect will be reduced by both post-trauma imaginal exposure and autobiographical memory elaboration.

*Hypothesis 5 (Intervention effects on reexperiencing)*: On the basis of the efficacy of prolonged exposure (Foa et al., 1999, 2005) and cognitive therapy (Ehlers et al., 2003, 2005) in the treatment of PTSD, we expected that both post-trauma imaginal exposure and autobiographical memory elaboration will lead to a reduction in reexperiencing symptoms following the experiment.

## 2 Method

### 2.1 Overview

Volunteers watched the analogue “traumatic” and neutral picture stories developed by Michael and Ehlers (2007). They were then randomly allocated to 20 min of either imaginal exposure, memory elaboration, or a control condition designed to prevent processing of the picture stories. A blurred picture task then tested perceptual priming and conditioned emotional evaluations for objects from the stories. To test the possible influence of explicit memory on the pattern of findings, participants also did a recognition test. Intrusive memories were assessed by telephone interview 4 weeks and 3 months later.

### 2.2 Participants

Participants were 122 volunteers who were recruited by advertisements in the local area in south London and via emails to staff and students of King’s College London. They were reimbursed with £15 for their time and travel expenses. Volunteers were excluded if they reported a history of trauma, current blood/injury phobia or severe depression. Table 1 shows participant characteristics. The experimental groups did not differ in sex, *χ*^2^ (2, 122) = 0.34, *p* = 0.844, age, *F* (1, 120) = 0.98, *p* = 0.378, trait anxiety, *F* (1, 121) = 1.50, *p* = 0.228 or state anxiety, *F* (1, 121) = 1.72, *p* = 0.182.

## 3 Picture stories

The experimental software for all parts of the experiment was programmed with SuperLab. Picture stories and memory tests were presented on a 15’’ screen of an Apple Macintosh computer. The picture stories were the same as in Michael and Ehlers (2007). Participants saw eight analogue “traumatic” and eight neutral picture stories, each consisting of three pictures. One additional neutral story (of a man getting drunk) was used to familiarize the participants with the task. The first picture (presented for 20 s) introduced the main character. It was always neutral so that it was not possible to conclude from this picture whether the story was unpleasant or neutral. The first picture contained two neutral objects in the background that were unimportant for the course of the story (*preceding* objects). The second picture (presented for 20 s) depicted the plot of the story and showed something traumatic or neutral happening. It contained one *central* object that was important for the course of the story, and determined whether the content was traumatic or neutral. The third and last picture (presented for 15 s) showed the final outcome for the main character of the story. It focused on and underlined the traumatic versus neutral content of the story. The unpleasant and neutral picture stories were matched for the number of males, females, and objects occurring in them, and whether the event happened indoors or outdoors. For example, one unpleasant story contained a dog killing a man and the matching story depicted a cat stealing a sandwich from its owner. A list of the content of all stories is presented in the Appendix. Table 2 illustrates the structure of one unpleasant (a man being stabbed and decapitated) and one neutral picture story (a man coming home and seeing his wife repairing a boot on the dining table), and lists what objects occur in these stories.

Participants were told that it was the purpose of the experiment to test how pictures affect people’s emotions. They were asked to watch the pictures closely and to imagine that they were present at the scene. After each picture story, they were asked to rate the pictures for pleasantness and arousal. There was no indication that memory for the pictures would be tested later.

Picture stories were presented in two blocks of “traumatic” and neutral stories, in counterbalanced order. Blockwise presentation was chosen to prevent crossover of negative emotions produced by unpleasant picture stories to neutral ones. Order of presentation did not influence the results. Between blocks participants had a 5-min break. The order of the stories within each block was randomized and different for every participant.

### 3.1 Experimental manipulation

Participants were randomly allocated to one of three experimental conditions that followed the picture story task.

#### 3.1.1 Exposure group

Instructions were modeled on imaginal exposure (Foa & Rothbaum, 1998). Participants were asked to close their eyes and to visualize the picture stories in their mind’s eye as vividly as they could. They were encouraged to visualize as much detail as possible and recall their own feelings and thoughts while holding the images in their mind. They were asked to go through the picture stories in the order they had seen them and to slow down when they came to the ones that they found most disturbing, making sure that they visualized all the details and recalled their feelings and thoughts at the time. The condition lasted 20 min. As a manipulation check, participants were asked every 5 min (via a tape recording) to write down what they had visualized and to rate how vivid the imaginal exposure had been, on a scale from 0 ‘not at all’ to 10 ‘very much’. The content of the notes indicated that participants had followed the instructions. The mean vividness rating was *M* = 7.20, SD = 1.75.

#### 3.1.2 Autobiographical memory elaboration group

This group received the same instructions as in Michael and Ehlers (2007). Participants were asked to answer five questions in writing, which were designed to facilitate autobiographical memory elaboration. The participants received a sheet of paper with the five questions, as well as oral instructions. The first question asked what the participants had done before they came to the experiment and how they had felt. The second question was whether the experiment had matched the participant’s expectations. In the third and fourth questions the participants were asked to think back to the stories and to indicate which ones they liked/disliked most, and whether the stories reminded them of things that had happened in their own lives. The final question asked about any plans that participants had for the rest of the day and how they felt about them. The experimenter reassured the participants that their answers would be treated with the strictest confidentiality. The participants had 20 min to answer the questions, and were instructed to spend the same amount of time on each of the questions. When the time for one question was up, participants were told to move on to the next question. The content of the notes indicated that participants had followed the instructions.

#### 3.1.3 Control group

Participants in the control group were told that the experiment dealt not only with the processing of pictures but also with the processing of words. They were given several sheets that each contained a different word task, and were told that they should try to complete as many of these tasks as possible in the next 20 min. The first task contained a list of neutral words and participants had to explain the meaning of these words (e.g. ‘connect’ means ‘join together’). The second task contained phrases, and participants had to identify words that were not correctly spelled given the context of the phrase (‘a honey be’). The third task was to identify which word of a group of four was misspelled (investigation, *readilly*, examined, assuming). The fourth task comprised groups of words, and participants had to find the word that was closest in meaning to a word typed above the group (malaria: basement, fever, theatre, fruit, ocean, tune). No task contained trauma-related words.

### 3.2 Memory measures

Perceptual priming for objects shown in the picture stories was tested with a blurred object identification task. To check for possible influences of explicit memory on the pattern of results, participants also completed a recognition test. In designing the memory tests, objects from the picture stories were isolated and edited using Adobe PhotoShop. All objects were left in their original size. As each participant completed both memory tests, two equivalent sets (set 1, set 2) of objects were created for each task. Each set contained one of the preceding objects from each story and, in the recognition task, half of the central objects from unpleasant and neutral scenes. Half of the participants saw objects from set 1 in the perceptual priming task and objects from set 2 in the recognition task. For the other half of the participants, sets were reversed. Picture set did not influence the results.

#### 3.2.1 Perceptual priming task

A blurred object identification task assessed priming as visual perceptual priming leads to an enhanced identification rate for previously seen objects. In order to decrease the chance that participants noticed that the task was a memory test, only the preceding objects from the stories (8 from “traumatic” and 8 from neutral stories) were included. Furthermore, the majority of items (*n* = 24) were unprimed objects that had not featured in the picture stories (e.g., a hole punch, scissors). These unprimed objects were matched for size to the primed objects from the picture stories.

Participants were told that they were now doing a different task that was unrelated to the picture stories. They were informed that the task was about how easy it is for people to identify blurred pictures. They were instructed to look at the pictures and guess what the object might be, working as quickly and as accurately as possible. The experimenter wrote down the answers, which were later coded for accuracy. If participants could not guess what the object might be, they indicated that they did not know. After their answer, participants moved on to the next object by pressing the space bar.

The preceding objects from the picture stories were blurred with a Gaussian filter to a degree which allowed approximately 50% correct identification in pilot participants with no prior exposure to the picture stories. The preceding objects shown in the “traumatic” and neutral stories did not differ in baseline identification rates (objects from unpleasant stories: *M* = 51.3%, objects from neutral stories: *M* = 49.8%, *N* = 40 pilot participants, Michael & Ehlers, 2007). The unprimed items were blurred slightly less so that their baseline identification rate was *M* = 59.7%. This was done to ensure that each participant would identify at least a few unprimed objects, thus reducing the chance that participants would notice that the task tests performance for stimuli from picture stories. Michael and Ehlers (2007) presented a series of pilot studies showing that there were no differences in priming, recognition memory or emotional evaluations when the objects were presented without their emotional context. Thus, possible memory differences in the present experiment can be interpreted as due to the emotional character of the stories.

The objects were presented on the computer screen in successive, random order that varied with each participant.

As the baseline identification rates for the individual objects varied substantially, we calculated the incremental identification probability for each object compared to its baseline identification rate. This method allowed us to obtain for each object a precise measure of how much its identification rate was increased through prior exposure. If the baseline identification rate for an object were, for example, 0.4, then the incremental identification rate would be 0.6 in the case of correct identification and −0.4 in the case of non-identification. For objects from picture stories, perceptual priming from prior exposure would show in positive incremental identification probability, whereas unprimed objects (no prior exposure) should be identified at baseline rates, resulting in a mean incremental identification probability of 0.

#### 3.2.2 Evaluative conditioning

To assess evaluative conditioning, participants rated how much they liked each picture of the blurred picture task on a scale from 0 ‘not at all’ to 100 ‘very much’, with 50 representing ‘neutral.’ Michael and Ehlers (2007) had shown that participants who had only seen the first picture of the picture stories rated the objects as neutral (objects from trauma stories: *M* = 45.20, SD = 12.77, objects from neutral stories, *M* = 44.56, SD = 12.32; distractor objects, *M* = 44.37, SD = 11.23).

#### 3.2.3 Recognition task

The recognition task tested explicit memory performance for objects from the picture stories. It included both central and preceding objects from the picture stories. For each “old” object from the picture stories, a parallel new object was chosen that looked somewhat different in appearance. These parallel new objects matched the objects from the picture stories in size and object type (e.g., if the object from the picture story was a watch, another watch of approximately the same size was used as the parallel object). Objects were presented on a computer screen in successive, random order which was different for each participant. Participants were asked to indicate whether or not they had seen the object previously in the stories by pressing the corresponding keys on the computer keyboard.

### 3.3 Self-report questionnaires

The Past Experience Questionnaire screens participants for a trauma history, blood/injury phobia, and severe depression. Participants who met any of these criteria were excluded from the study. Participants also completed the state and trait versions of the State-Trait Anxiety Inventory (Spielberger, Gorsuch, Lushene, Vagg, & Jacobs, 1983) and a 4-item Mood Questionnaire comprising 4 Likert scales assessing current mood (happy, anxious, depressed, angry), each on a scale from 0 ‘not at all’ to 100 ‘extremely.’ The total score (with happy ratings reversed) was used for data analysis.

### 3.4 Intrusive memories interview

At 4 weeks and 3 months, participants were interviewed on the telephone. They were asked about any unwanted memories of the picture stories that had occurred without an apparent reason. They rated the frequency of such memories in the past 2 weeks on a scale from 0 ‘never’ to 7 ‘more often than once a day’.

### 3.5 Procedure

The study was approved by the local Ethics Committee. Participants received an information sheet about the study and were given further information on the telephone when arranging the appointment. They were informed in writing and by the experimenter that the study involved watching some unpleasant pictures and that they could withdraw at any time without having to give a reason. On arrival at the laboratory, participants gave written consent. They then completed the Past Experience Questionnaire, the STAI (state version) and the first Mood Rating. Participants were then given oral and written instructions for watching the picture stories, and watched the two blocks of picture stories. They completed a Mood Rating after each of the blocks. The experimental manipulation (20 min) followed. Participants completed another Mood Rating and then had a 10-min break, during which the experimenter served a drink and conversed with them about unrelated matters. They then completed the perceptual priming task and the emotional evaluation ratings, followed by completion of the STAI (trait version) and the object recognition task.

The experimenter made sure that participants felt well before leaving and gave participants her contact details, encouraging them to get in touch if they felt in any way distressed about the experiment. However, none of the participants took up this offer and none reported that they found the experiment too distressing.

Participants completed the Intrusive Memories Interview over the telephone at 4 weeks and 3 months after the experiment. At 3 months they were debriefed about the purposes of the study.

### 3.6 Data analysis

#### 3.6.1 Perceptual priming task

The priming index was computed to compare priming for objects from “traumatic” versus neutral stories. The priming index was the difference between the incremental identification rates for primed minus unprimed objects. Greater scores indicate greater priming.

#### 3.6.2 Emotional evaluations

A parallel evaluative conditioning index was calculated as the difference between the emotional evaluations for the primed minus the unprimed objects. Negative scores indicate greater conditioned emotional evaluations.

#### 3.6.3 Object recognition task

Data analysis followed signal detection theory (SDT) (MacMillan & Creelman, 1991). From the hits (correct recognition of original objects) and false alarms (erroneous recognition of parallel objects), sensitivity (*d*’) and response bias (*c*) scores were calculated, for both preceding and central objects. Sensitivity is a standard measure of recognition memory performance that measures how well participants discriminated between objects from the stories and parallel objects that they had not seen before, calculated as *d*’ = probit (hits) − probit (false alarms). Response bias is a measure of leniency in endorsing an object as “old”, calculated as *c* = −0.5*(probit (hits) + probit (false alarms)).

#### 3.6.4 Statistical analysis

Data were analyzed with the General Linear Models (GLM) and CROSSTABS procedures in SPSS 15.0. Planned contrasts were used to test the hypotheses. Greenhouse–Geisser corrections were used if appropriate. We report one-tailed significance levels for planned contrasts as the hypotheses were uni-directional, and two-tailed significance levels for other comparisons. Details of the analyses are found below.

## 4 Results

### 4.1 Perceptual priming task

In no case did participants falsely identify an object from the picture stories when another object was presented.

#### 4.1.1 Did priming occur?

To test whether watching the picture stories led to perceptual priming, incremental identification rates for the objects from picture stories (primed objects) were compared with those of matched distracter items that participants had not seen before (unprimed objects). Primed objects from picture stories were identified with greater probability than unprimed objects without prior exposure. *M* = 0.02, SD = 0.14 versus *M* = −0.03, SD = 0.18, *F* (1,121) = 11.88, *p* < 0.001, *η*^2^ = 0.089.

#### 4.1.2 Hypothesis 1 (Enhanced perceptual priming)

**Fig. 1:**
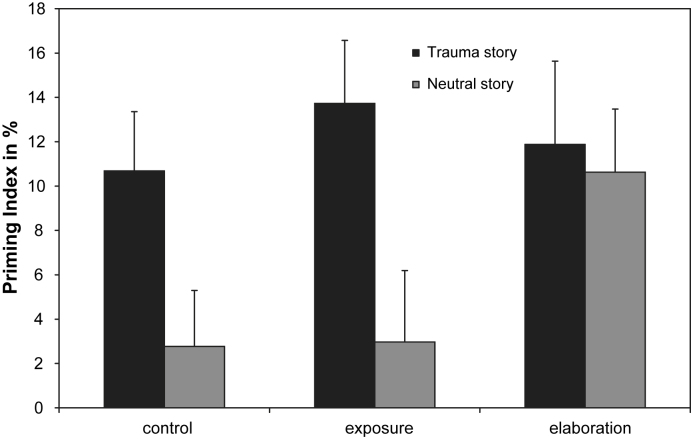
Group differences in the priming index (enhancement of identification probability in percent) for neutral objects previously seen in the context of traumatic or neutral picture stories. The groups received different post-exposure interventions (control task without memory processing, imaginal exposure, autobiographical memory elaboration).

The results for the perceptual priming task (priming index) are presented in Fig 1. A repeated measures ANOVA showed a significant main effect of story context (traumatic versus neutral), *F*(1,121) = 9.29, *p* = 0.003, *η*^2^ = 0.071. Consistent with the enhanced priming hypothesis, blurred pictures of neutral objects that had preceded the “traumatic” pictures in the picture stories were more readily identified, *M* = 0.12, SD = 0.19, than those that preceded neutral pictures, *M* = 0.05, SD = 0.19.

#### 4.1.3 Hypothesis 2 (Effects of elaboration on enhanced priming)

The planned contrast comparing the enhanced priming effect (difference between the priming indices for “traumatic” and neutral stories) for the autobiographical memory elaboration group with the other two groups was significant, *F* (1,119) = 3.03, *p* = 0.042, *η*^2^ = 0.025. Enhanced priming for objects from “traumatic” stories was evident in both the control, *F*(1,40) = 5.15, *p* = 0.029, *η*^2^ = 0.114, and the imaginal exposure conditions, *F*(1,40) = 6.93, *p* = 0.012, *η*^2^ = 0.148, whereas the autobiographical memory elaboration group showed equal priming for objects from “traumatic” and neutral picture stories, *F*(1,39) = 0.11, *p* = 0.740, *η*^2^ = 0.003.

#### 4.1.4 Hypothesis 3 (evaluative conditioning effect)

**Fig. 2:**
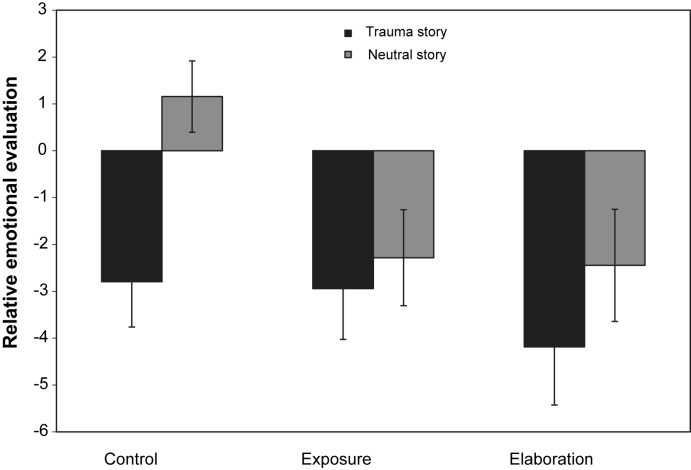
Group differences in conditioned evaluations for neutral objects previously seen in the context of traumatic or neutral picture stories. Positive numbers reflect a more positive evaluation. The groups received different post-exposure interventions (control task without memory processing, imaginal exposure, autobiographical memory elaboration).

The results of the emotional evaluations of the objects from the picture stories are presented in Fig. 2. A repeated measures ANOVA showed a significant effect of story context (traumatic versus neutral), *F*(1,121) = 8.44, *p* = 0.004, *η*^2^ = 0.065. As expected, neutral objects that had preceded “traumatic” pictures were evaluated more negatively, *M* = −3.30, SD = 7.00, than those that preceded neutral pictures, *M* = −1.18, SD = 6.58.

#### 4.1.5 Hypothesis 4 (Intervention effects on evaluative conditioning)

The planned contrast comparing the conditioned evaluation effect (difference between emotional evaluations of objects from “traumatic” and neutral stories) for the two intervention groups and the control group was significant, *F* (1,119) = 3.21, *p* = 0.038, *η*^2^ = 0.026. Participants in the control group showed the evaluative conditioning effect and gave more negative emotional evaluations for objects from traumatic stories, *F*(1,40) = 13.85, *p* = 0.001, *η*^2^ = 0.257. This was not the case for the two intervention groups, imaginal exposure, *F*(1,40) = 0.30, *p* = 0.587, *η*^2^ = 0.007; autobiographical memory elaboration group, *F*(1,39) = 1.39, *p* = 0.245, *η*^2^ = 0.034.

### 4.2 Intrusion interviews

The planned contrast testing Hypothesis 5 (intervention effect on unwanted memories) showed the expected difference between the intervention groups and the control group in the frequency of intrusive memories at 4 weeks, *F* (1,118) = 3.46, *p* = 0.032, *η*^2^ = 0.029. The imaginal exposure, *M* = 0.46, SD = 0.87, and autobiographical memory elaboration groups, *M* = 0.56, SD = 1.27, reported fewer unwanted memories than the control group, *M* = 1.02, SD = 1.93. At 3 months, very few participants reported unwanted memories, but the intervention groups were still less likely to report unwanted memories, 14.3% of the control group and 5.2% of the intervention groups, *χ*^2^ (1, *n* = 112) = 2.69, *p* = 0.05.

### 4.3 Further analyses

#### 4.3.1 Recognition memory

The results of the object recognition task are presented in Table 1. Sensitivity and response bias were analyzed using 3 × 2 × 2 GLMs, with experimental group as the between subject factor and story context (“traumatic” versus neutral) and object importance (central versus preceding) as within-subject factors. There was no main effect, *p* > 0.41, nor interactions, all *p* > 0.14, with experimental group. As to be expected on the basis of eye-witness research (Christianson, 1992), there was a main effect of object importance; central objects were better discriminated than preceding objects, *F* (1, 119) = 176.63, *p* < 0.001, *η*^2^ = 0.600. In contrast to the perceptual priming task, there was no main effect of story context (“traumatic” versus neutral) in the sensitivity with which the objects were identified in the recognition task, *F*(1, 119) = 0.91, *p* = 0.341, *η*^2^ = 0.008, There was an interaction between story context and object importance, *F* (1, 119) = 6.48, *p* = 0.012, *η*^2^ = 0.052. Post-hoc analyses showed that this interaction was due to somewhat better discrimination of central objects from neutral compared to central objects from “traumatic” stories, *F* (1, 121) = 4.21, *p* = 0.042, *η*^2^ = 0.034. There was no difference in the sensitivity of discriminating preceding objects from traumatic and neutral stories, *p* > 0.18.

Table 1 also presents the results of response bias analyses. There was no main effect, *p* > 0.69, nor interactions, all *p* > 0.16, with experimental group. There was a main effect of story context, *F* (1, 119) = 9.01, *p* = 0.003, *η*^2^ = 0.071, which was qualified by a trend for an interaction between story context and object importance, *F* (1, 119) = 2.81, *p* = 0.096, *η*^2^ = 0.023. Further analyses showed that participants used a more liberal response criterion for central objects from “traumatic” stories than for central objects from neutral stories, *F* (1, 119) = 8.04, *p* = 0.005, *η*^2^ = 0.063. However, there was no effect of story context for the preceding objects, *F* (1, 119) = 1.94, *p* = 0.167, *η*^2^ = 0.016.

Sensitivity and response bias for preceding objects from “traumatic” stories in the recognition task did not correlate with identification rates in the perceptual priming test, *r* = 0.10, *p* = 0.289, and, *r* = 0.08, *p* = 0.387, respectively.

#### 4.3.2 Mood ratings

A 3 × 4 GLM with experimental group as the between subject factor and experimental phase (before picture stories, after neutral stories, after trauma stories, after experimental manipulation) as the within-subject factor showed significant effects of experimental phase, *F* (3, 354) = 34.13, *p* < 0.001, *η*^2^ = 0.225, and a group × phase interaction *F* (3, 354) = 3.66, *p* < 0.003, *η*^2^ = 0.058. Separate group comparisons for each experimental phase showed a significant group difference in negative mood after the experimental manipulation, *F* (1, 118) = 4.77, *p* = 0.010, *η*^2^ = 0.075, but not for any of the other time points, all *p*s > .23. The imaginal exposure group reported more negative mood after the experimental manipulation than the control group, *p* = 0.003. The memory elaboration group did not differ significantly from the other two groups.

## 5 Discussion

This study was motivated by two clinical observations. First, intrusive trauma memories often appear to be triggered by cues that are perceptually similar to the intrusions, or to stimuli that immediately preceded the respective sensation during the trauma (e.g., Ehlers & Clark, 2000; Ehlers et al., 2002, 2004). Second, a range of therapeutic interventions including imaginal reliving (Foa & Rothbaum, 1998) and cognitive interventions designed to increase the elaboration of the traumatic experience and link it with its context of other autobiographical information (Ehlers & Clark, 2000) are effective in reducing such unwanted memories. The study explored whether priming for stimuli that occur in the context of trauma and conditioned emotional evaluations may play a role in these therapeutic effects.

The two memory mechanism under investigation showed the expected pattern of differences between objects that were perceived in the context of trauma and those that occurred in neutral contexts. In line with Hypothesis 1 (enhanced priming effect), the study replicated previous results (Arntz et al., 2005; Ehlers et al., 2006; Michael & Ehlers, 2007) that neutral objects that occur in a “traumatic” context are more strongly primed than comparable stimuli that occur in a neutral context. The results complement those demonstrating that people with PTSD show stronger priming for trauma-related sentences or words than trauma survivors without PTSD (Amir et al., 1996; Michael, Ehlers, & Halligan, 2005). Enhanced priming was experimentally induced by embedding neutral objects in a context of either “traumatic” or neutral picture stories. When presented without this context, the degree of priming for the objects from these scenes did not differ (Michael & Ehlers, 2007). Thus, the differences in priming can be attributed to the different emotional context in which the objects occurred. Perceptual priming may be more influenced by stimulus context and possibly interacts more closely with conditioning processes than previously thought (e.g., Squire, 2004). Overall, the results appear to be consistent with theories that emphasize the role of associative learning in perceptual learning (e.g., McLaren & Mackintosh, 2000).

There has been a debate in the literature on the influence of explicit memory on the performance in implicit memory tasks such as priming tests (e.g., Jacoby, Toth, & Yonelinas, 1993; Tulving, Schacter, & Stark, 1982). The present study was not designed to address this issue so we cannot conclude with certainty that a pure implicit memory effect was observed. However, the pattern of findings in the recognition test makes it very unlikely that the enhanced priming effect for objects from the “traumatic” stories stemmed from the intentional search for these objects and the use of explicit knowledge. On the basis of eye-witness research (e.g., Christianson, 1992), one may have expected enhanced sensitivity in the recognition test for central objects from “traumatic” stories compared to central objects from neutral stories. This was not the case in this experiment, possibly due to ceiling effects as all participants showed very good sensitivity in recognizing central objects. Importantly, there was no indication of an enhanced sensitivity in recognizing preceding objects from traumatic stories. Furthermore, previous research would suggest that if emotional context has an effect on preceding stimuli, it would be in the direction of poorer rather than better recall (Christianson, 1992). Thus, it is extremely unlikely that the enhanced priming effect for these stimuli can be explained by enhanced explicit memory.

In line with Hypothesis 3 (*evaluative conditioning effect*), participants reported that they liked neutral objects that had preceded the “traumatic” pictures less than those that had preceded neutral pictures. These results are in line with the general literature on evaluative conditioning (Hofmann et al., 2010). They further show that evaluative conditioning can take place even with a single pairing of the conditioned stimulus (preceding object) and unconditioned stimulus (“traumatic” picture).

The experimental interventions used for the study were modeled on treatment procedures used in effective treatments of PTSD. As expected (Hypothesis 5, intervention effect on unwanted memories), both interventions decreased the likelihood of subsequent intrusive memories. This result is in line with the effects of imaginal exposure (Foa, Zoellner, & Feeny, 2006) and cognitive therapy as early interventions for PTSD (Ehlers et al., 2003). They suggest that such very early post-trauma interventions may have a role in preventing unwanted memories of the trauma and, possibly, PTSD. The interventions studied here complement other research on the possible prevention of reexperiencing symptoms. While some clinically-derived post-trauma interventions such as psychological debriefing have not shown promising results (e.g., Rose, Bisson, & Wessely, 2003), interventions that are based on cognitive science may show greater promise (e.g., Holmes, James, Coode-Bate, & Deeprose, 2009; Kindt, Soeter, & Vervliet, 2009).

The effects of the interventions on the two memory mechanisms under investigation differed, as expected. In line with Hypothesis 2 (effects of elaboration on enhanced priming), a post-“trauma” intervention designed to increase the elaboration of the picture stories in the context of the participants’ other autobiographical experiences eliminated the enhanced priming effect. This result replicates Michael and Ehlers’s (2007) findings. The present study extended the previous results in two respects. First, the study showed that imaginal exposure does not eliminate the enhanced priming effect and thus operates via other mechanisms. Second, the study clarified that memory elaboration appears to operate by enhancing priming for neutral stimuli stimuli rather than by decreasing priming for stimuli from the traumatic context. This finding may have therapeutic implications as it highlights the need to increase awareness of signs that the world is has gone back to normal and is reasonably safe after traumatic experiences. This may be promoted in several ways, for example, stimulus discrimination training to promote awareness that the current environment is different to the trauma, or reclaiming your life assignments to promote access of memories of the non-traumatized self before the trauma (Ehlers & Clark, 2000; Ehlers et al., 2004).

In line with Hypothesis 4 (intervention effects on evaluative conditioning), both interventions affected emotional evaluations of the stimulus material from the picture stories. In contrast to the control group who reported to dislike the stimuli from the “traumatic” picture stories more than those from the neutral stories, and thus showed the expected conditioned evaluation effect, participants who had received the interventions gave similar ratings regardless of the context in which they had experienced the objects. This demonstrates that conditioned evaluations can be modified in several ways. The lack of differential ratings to objects from the trauma stories may reduce the probability that negative mood is triggered by similar cues in the environment, which may then trigger trauma memories. It is of interest to note that the intervention groups did not give neutral ratings. Rather, they reported that they disliked objects from *both* sets of stories. The findings parallel research into learned fear responses showing that learned associations are not erased by subsequent extinction training, but can be inhibited (LeDoux, 1992). Both the results for perceptual priming and evaluative conditioning suggest that participants’ responses to the objects from the “traumatic” stories were relatively unaffected by the interventions. Rather, the interventions facilitated similar responses to the objects from neutral stories. Thus, both interventions may prevent selective responses to trauma reminders in the natural environment.

The interventions used in this experiment delivered components of complex treatment protocols without the context of a therapeutic relationship to study their pure effects. The pattern of results may be therefore be different for the full treatment programs. For example, the imaginal exposure condition used in this study did not affect priming. This procedure used the visualization component of the *Prolonged Exposure* treatment protocol (Foa & Rothbaum, 1998) and participants did not give a running commentary about what they were visualizing and did not discuss their experience afterwards. It is possible that an effect on priming would have been observed if participants had verbalized their experience. This would be in line with theories that emphasize the role of creating a trauma narrative in PTSD (Brewin et al., 1996; Foa & Riggs, 1993). Similarly, the memory elaboration condition did not include methods to facilitate access to the most traumatic moments and their meanings that are used in *Cognitive Therapy for PTSD* (Ehlers et al., 2003, 2005), nor other techniques to change problematic meanings such as socratic questioning or behavioral experiments. Including these techniques may have enhanced the effects of the intervention.

It is noteworthy the term memory elaboration as used in this study differs from the way it is interpreted by Rubin, Berntsen, and Bohni (2008). This study followed Ehlers and Clark’s (2000) and Ehlers et al. (2004)’s hypothesis that a predominance of data-driven (as opposed to conceptual) and a lack of self-referential processing during the trauma leads to a relatively poor elaboration of trauma memories, in particular, a failure to link the worst moments of traumatic experience with other relevant information in autobiographical memory. This is thought to prevent these moments from being updated with other relevant (usually subsequent) information that corrects predictions made at the time such as “I did not die,” “I saw my children again.” Rubin et al. (2008) on the other hand suggested an enhanced elaboration of the trauma in that it is thought to become a central component of identity and reference point for other autobiographical experiences. These hypotheses are not mutually exclusive. Several studies showed that both memory disorganization and appraisals of being permanently changed by the trauma predict PTSD (e.g., Dunmore, Clark, & Ehlers, 2001; Ehlers, Maercker, & Boos, 2000; Halligan, Michael, Clark, & Ehlers, 2003). The permanent change concept is very similar to Rubin et al.’s (2008) centrality of event concept.

The study had several limitations. First, the experiments used an analogue design and it remains unclear to what extent the results would generalize to traumatic events that would meet DSM-IV (American Psychiatric Association, 1994) criteria. Ethical considerations limit the induction of trauma in the laboratory. Similarly, it remains uncertain to what extent the unwanted memories that participants reported in this study are comparable to those in PTSD, although it is known that witnessing horrific events can be sufficiently traumatic to induce PTSD (American Psychiatric Association, 1994). Second, the study only assessed two memory processes and other processes are likely to play a role in reexperiencing symptoms, e.g. Pavlovian conditioning of physiological responses (e.g., Foa et al., 1989). Third, there was considerable interindividual variability and the effect sizes for the effects of the intervention were small. In particular, the proportion of participants who experienced intrusive memories of the picture stories was low. This reduced the power of the analyses and possibly the effect sizes. It is possible that clearer group differences would have emerged if intrusions had been assessed somewhat earlier, for example, at 2 weeks after the experiment. Fourth, the stimulus material had some limitations. The results are based on a relatively small number of objects. In order to make the material as realistic as possible, the paradigm used film clips that show the main character from the story throughout the picture story. This made it impossible to counterbalance the objects across the neutral and “traumatic” picture stories. Although the objects were carefully matched for ease of identification, salience and memorability and Michael and Ehlers’ (2007) data consistently showed that the objects were comparable in these respects, we cannot completely rule out that subtle material effects influenced the results. It is therefore reassuring that a recent study (Sündermann, Hauschildt, & Ehlers, in preparation) replicated the enhanced priming effect when objects were counterbalanced across neutral and trauma stories.

## Figures and Tables

**Table 1:** Sample characteristics and control variables.

	Control group	Imaginal exposure group	Memory elaboration group
Sex male (*n*, %) female (*n*, %)	20 (49%) 21 (51%)	18 (44%) 23 (56%)	20 (50%) 20 (50%)
Age *M* (SD)	32.38 (10.87)	30.80 (11.45)	29.13 (8.56)
STAI – state anxiety *M* (SD)	30.73 (6.93)	32.19 (7.98)	29.23 (6.60)
STAI – trait anxiety *M* (SD)	38.93 (9.37)	35.61 (7.95)	37.05 (8.73)

Negative mood *M* (SD)			
before picture stories	13.25 (7.41)	14.15 (9.06)	13.38 (8.69)
after neutral stories	17.47 (12.29)	18.17 (13.65)	14.84 (8.27)
after trauma stories	27.63 (18.18)	23.90 (16.84)	22.31 (12.12)
after experimental manipulation	12.59 (9.47)	20.67 (13.16)	16.78 (12.33)

Recognition test Discrimination sensitivity *d*’			
central objects/trauma stories	2.38 (1.81)	3.01 (1.85)	2.42 (1.68)
central objects/neutral stories	2.91 (1.69)	2.95 (1.87)	3.24 (1.68)
preceding objects/trauma stories	1.10 (1.06)	1.48 (1.44)	1.12 (1.26)
preceding objects/neutral stories	1.06 (1.10)	1.09 (0.93)	1.01 (1.36)

Response criterion			
central objects/trauma stories	0.02 (0.84)	0.34 (0.94)	0.27 (0.75)
central objects/neutral stories	0.52 (1.04)	0.55 (0.96)	0.46 (0.94)
preceding objects/trauma stories	0.38 (0.56)	0.34 (0.94)	0.39 (0.56)
preceding objects/neutral stories	0.42 (0.65)	0.34 (0.66)	0.63 (0.55)

**Table 2:** Story structure, example of one “traumatic” and one parallel neutral story. The objects for which perceptual priming and recognition memory were later tested are in italics.

	“Traumatic” story	Neutral story
PICTURE 1: Main character in neutral setting	A man watching TV	A man entering the kitchen
*preceding stimuli (blurred picture and recognition tests)*	*bottle, cushion*	*spatula, frying pan*
PICTURE 2: Main character experiences “traumatic” or neutral event	The man is attacked with a knife by an intruder	The man notices that his wife is repairing an old boot on the kitchen table
*central stimuli (recognition test only)*	*knife*	*boot*
PICTURE 3: “Traumatic” versus neutral outcome	Decapitated man	Puzzled man

## Appendix Contents of the picture stories

*Practice story:*

1. A man getting drunk

*“Traumatic” stories*:

1. An execution
2. A woman being raped by a man
3. A man being drowned in a bath tub
4. A dog killing a man
5. A plane crash
6. A woman being strangled by a man
7. A warehouse owner being robbed and tortured
8. A man being stabbed and decapitated

*Neutral stories*:

1. Teenagers discussing basketball
2. Couple snuggling up in bed together
3. A nun welcoming a couple to a nursing home
4. A cat stealing its owner’s sandwich
5. House move
6. A woman making a telephone call
7. A man coming home and seeing his wife repairing a boot on the dining table
8. A worker washing himself at the end of his shift
